# *Brassica oleracea* Var *italica* by-Products Prevent Lipid Accumulation and Cell Death in a Liver Cell Model of Lipid Toxicity

**DOI:** 10.3390/nu15040924

**Published:** 2023-02-12

**Authors:** José P. Castelão-Baptista, Sara A. Valente, Sara Canário, David Oppolzer, Ana Barros, Carlos Venâncio, Tânia Martins, Luís Antunes, Vilma A. Sardão, Eduardo Rosa, Paulo J. Oliveira

**Affiliations:** 1 CNC—Center for Neuroscience and Cell Biology, UC-Biotech, University of Coimbra, 3060-197 Cantanhede, Portugal; 2 Centre for the Research and Technology of Agro-Environmental and Biological Sciences (CITAB), University of Trás-os-Montes and Alto Douro (UTAD), 5000-801 Vila Real, Portugal; 3 Inov4Agro—Institute for Innovation, Capacity Building and Sustainability of Agri-Food Production, University of Trás-os-Montes and Alto Douro (UTAD), 5000-801 Vila Real, Portugal

**Keywords:** phytochemicals, NAFLD, HepG2, natural products, FFA

## Abstract

Obesity, a rising concern in the Eastern world, encompasses several co-morbidities, namely non-alcoholic fatty liver disease (NAFLD). Potential natural-based interventions to decrease the burden of obesity complications are being investigated. Many of the edible parts of plants are not sold for consumption and end up as massive waste, losing nutritional potential. In fact, a sizeable amount of waste is generated within the different steps of the food supply chain, representing a massive loss of both plant material and natural resources. A good example is *Brassica* by-products (BBPs). The objective of this work was to investigate the effect of three different extracts from broccoli (*Brassica oleracea* var *italica*) by-products in an *in vitro* model of free fatty acid (FFA)-induced lipotoxicity using human hepatoma HepG2 cells. Broccoli leaf, stalk, and inflorescence extracts induced a dose-dependent decrease in the cell viability of HepG2 cells. However, the maximal non-lethal concentrations of leaves, stalks, and inflorescences (10 μg/mL) did not compromise mitochondrial function or neutral lipid accumulation in HepG2 cells. The extracts significantly decreased FFA-induced lipid accumulation in HepG2 cells either in a co-incubation or pre-incubation strategy. The broccoli extracts’ capacity to prevent the FFA-induced decrease in catalase activity in HepG2 may explain the observed effects.

## 1. Introduction

Obesity is a substantial public health concern with increasing social and economic costs [1]. Although the WHO does not have more recent or comprehensive data, it was estimated that in 2016, about 39% of the world’s population (around 1.9 billion adults) was overweight, and of these, around 32% (600 million) were obese [2]. These numbers have tripled since 1975, indicating a troubling upward trend [2]. Most cases of obesity are due to excessive energy consumption from dietary intake compared to the expense of energy through metabolism and physical activity. In the remaining cases, various factors such as environmental, genetic, behavioral, and socioeconomic status interact to cause this condition [3,4].

Obese patients often suffer from comorbidities and metabolic disorders, including hypertension, coronary heart disease, type 2 diabetes, psychological problems, several cancers, and non-alcoholic fatty liver disease (NAFLD) [5,6,7]. NAFLD involves an excessive accumulation of fatty acids in the liver without significant alcohol consumption [8,9]. Given the global prevalence of obesity, it is no surprise that around 25.2% of the world’s population suffers from NAFLD, with the expectation that this number will hit 33.5% in 2030 [10,11]. The severity of NAFLD can vary from simple hepatic steatosis to non-alcoholic steatohepatitis (NASH) [12,13]. NAFLD can lead to cirrhosis and liver cancer [14]. Hepatocyte mitochondria up-regulate the β-oxidation pathway to counteract the excessive fatty acid influx, generating reactive oxygen species (ROS) as a by-product [8,9], ultimately triggering oxidative stress in later stages of the disease [15]. Mitochondria can also become impaired due to excessive triglyceride accumulation in the cytoplasm of hepatocytes in the form of fat droplets [16]. The accumulation may cause an increase in fat oxidation through the peroxisomal pathway, generating further excess ROS and disturbing peroxisomal redox homeostasis [17].

There is no approved therapy for NAFLD, especially for more advanced stages, such as NASH, despite numerous ongoing clinical trials. Several natural products are being tested to prevent and/or delay NAFLD progression [18]. Natural products are a good source of bioactive compounds [19,20,21] and may constitute the basis of supplements or drugs for metabolic diseases. The biological potential of *Brassica oleracea* by-products (BBPs) against metabolic disruption in obesity is very well documented [22]. Within the varieties of *Brassica oleracea*, broccoli (*Brassica oleracea* var *italica*) has emerged as a potential candidate to aid in the treatment of obesity, essentially due to the large amount of glucosinolates it contains, namely glucoraphanin, the precursor of sulforaphane [23,24,25,26]. Well-known compounds within BBPs have antioxidant effects, such as phenols, glucosinolates, carotenoids, and ascorbic acid [27]. Despite being rich in bioactive compounds, plant by-products are, in essence, discarded since they are generally not used for human consumption; often, they include leaves, stalks, and processing waste [28]. In the case of broccoli (*Brassica oleracea* var *italica*), they represent 75% of the plant [29]. Annually, many tons of waste are produced since broccoli is one of the most cultivated crops around the world. Therefore, finding new medicinal uses for these BBPs could significantly impact return on investment, market price, and the environment due to the sheer amount of waste generated for a small portion of usable products [30].

We investigated here the medical potential of BBP extracts against steatosis and oxidative damage in human HepG2 cells, chosen as a biological model for hepatocytes, mimicking the hepatic pathophysiology observed in NAFLD. Our *in vitro* study shows the potential of BBP extracts in reducing lipid accumulation in hepatic cells subjected to supraphysiological concentrations of fatty acids. The remarkable effects highlight the therapeutic potential of BBPs for the prevention/treatment of NAFLD.

## 2. Materials and Methods

***Brassica oleracea* extracts preparation**—Plant extracts in powder form from leaves, stalks, or inflorescences, were suspended in dimethyl sulfoxide (DMSO) at a concentration of 50 mg/mL and kept at −20°.

Plant powder extracts were obtained from three broccoli plants randomly selected from 15 specimens collected for a larger study. These were collected from the producer Quinta do Celão, Unipessoal Lda, Campo Bolão-Adémia, Coimbra, from a crop field located in Quinta das Abertas, Condeixa-a-Nova, Coimbra, on October 22 of 2018, under fall season conditions. During harvest, entire plants were carefully removed from the soil, including their roots and soil, as much as possible, to extend the post-harvest lifetime under transportation to the laboratory, and then carefully placed in individual bags. Each plant was cut and divided into inflorescences, leaves, and stalks. The roots were discarded. Each part was then weighed and kept at −80 °C. The intact plant material was then freeze-dried and ground to a fine powder [31,32].

To extract polar phenolic compounds [33], samples (40 mg) were mixed with 1.5 mL of methanol/distilled water (70:30, *v*/*v*), vortexed, and then extracted for 30 min by agitation at room temperature (RT). The mixture was then centrifuged for 15 min at 10,000 rpm and 4 °C to separate the supernatants from the solid residue. The supernatants were kept in a volumetric flask of 5 mL. This extraction was performed in triplicate, and the supernatants from each extraction were mixed. With the aforementioned solvent, the final volume was 5 mL. The methanolic extracts were then filtered through 0.45 µm PVDF filters (Millipore, Bedford, MA, USA, Millex HV13).

**Quantitative Phytochemical Analysis and Antioxidant Activity**—The phenolic content and the antioxidant capacity were evaluated in broccoli plant extracts, namely leaves, stalks, and inflorescences. The content of total phenols, *ortho*-diphenols, and flavonoids was then determined by colorimetric assays as previously reported [33,34] using a 96-well microplate reader (Multiskan FC Microplate Photometer, Thermo Fisher Scientific, Vantaa, Finland). For the determination of total phenol content, 20 μL of each sample extract, 100 μL of diluted Folin–Ciocalteu reagent (10%, *v*/*v*), and 80 μL of aqueous sodium carbonate (7.5%, *w*/*v*) were mixed and then incubated at 42 °C for 30 min in the dark. The absorbance was measured at 750 nm, and the results were expressed as mg of gallic acid per gram of dry weight (mg gallic acid/g DW). The *ortho*-diphenols content was evaluated by mixing 160 μL of diluted extract with 40 μL of sodium molybdate solution (5%, *w*/*v*) prepared with hydro-methanol (50%, *v*/*v*). Mixtures were incubated at room temperature for 15 min in the dark. The absorbance was measured at 375 nm, and the results were expressed as mg gallic acid/g DW. For the assessment of flavonoid content, 24 μL of diluted extract was added to 28 μL of sodium nitrite (5%, *w*/*v*). After 5 min of incubation at room temperature, 28 μL of a 10% (*w*/*v*) aluminum chloride solution was added, and the mixture was left to react for 6 min. Then, 120 μL of sodium hydroxide (1 M) was added and the mixture was shaken for 30 s. The absorbance was read at 520 nm, and the results were expressed in mg of catechin per gram of dry weight (mg catechin/g DW). The free radical scavenging activity was determined by ABTS (2,2-azinobis (3-ethylbenzothiazoline-6-sulfonic acid) diammonium salt) and DPPH (2,2-diphenyl-1-picrylhydrazyl) assays as previously reported [35], with slight modifications. The measurements were performed at a microscale using a 96-well microplate reader (Multiskan FC Microplate Photometer, Thermo Fisher Scientific, Vantaa, Finland). In the ABTS assay, 12 µL of the samples were added to 188 µL of ABTS solution (20 mM), and in the DPPH assay, 10 µL of the samples were added to 190 µL of DPPH solution (8.87 mM). The absorbances were read at 734 nm after 30 min of reaction, at room temperature, for ABTS•+, and at 520 nm after 15 min of incubation for DPPH•. For both assays, blanks were made with the solvents (distilled water for ABTS and 70% (*v*/*v*) methanol for DPPH assay), whose absorbance values were subtracted from the sample absorbance. The results were expressed in mmol of Trolox per gram of dry weight (mmol Trolox/g DW).

For all quantitative phytochemical analyses and antioxidant activity, the hydromethanolic extracts of each part of the plant were diluted 10 times.

Fatty acid preparation—To prepare the fatty acid mixtures (FFAs) as saponified 10 mM stock solutions: Free fatty acid BSA (1:1) was complexed at 50 °C for 10 min. After cooling at room temperature, 0.2 g/mL of free fatty acid BSA was diluted in 25 mM KOH and used as a control. The same dilution ratios were used for the free fatty acid BSA control FA. The amounts used in the mixture FA were combined to mimic lipid accumulation in a high-calorie Western diet [36]: 39% C16:0 (catalog number: P0500, Sigma-Aldrich (Steinheim, Germany).; 5% C18:0 (catalog number: 85679, Sigma-Aldrich (Steinheim, Germany).); 50% C18:1 (catalog number: O1008, Sigma-Aldrich (Steinheim, Germany).; 4% C18:2 (catalog number: L1376, Sigma-Aldrich, USA); and 2% C20:4 (catalog number: A3611, Sigma-Aldrich (Steinheim, Germany).

**Cell culture**—Human hepatocellular carcinoma HepG2 cells (Catalogue 85011430, ECACC, UK) were cultured in a low-glucose medium composed of Dulbecco’s modified Eagle’s medium (DMEM; Catalogue D5030, Sigma-Aldrich, USA) supplemented with 5 mM glucose, sodium bicarbonate (3.7 g/L), HEPES (1.19 g/L), L-glutamine (0.876 g/L), sodium pyruvate (0.11 g/L), 10% fetal bovine serum (FBS), and 1% penicillin–streptomycin 100 x solution in a humidified atmosphere (5% CO_2_, 37 °C).

**Cell treatment**—HepG2 cells were seeded (6.0 × 10^4^ cells/cm^2^) and grown for 24 h, reaching 60–70 % confluence before treatment.

Cytotoxicity assessment in HepG2—Cells were treated with increasing concentrations of *Brassica oleracea* extracts (leaves, stalks, and inflorescences) for 24 or 48 h periods of time.

Hepatoprotective assessment in HepG2—*Brassica oleracea* extracts (leaves, stalks, and inflorescences at either 1 or 10 µg/mL) were pre-incubated for 24 or 48 h or co-incubated for 3 or 24 h following oxidative stress- or lipotoxicity-induced damage (details are provided in the Supplementary Materials, Table S1). Tert-butyl hydroperoxide (t-BHP; 100 µM, 3 h) was used for causing oxidative stress-induced damage, while a mixture of free fatty acids (FFAs; 250 µM, 24 h in cell culture medium with 1% FBS) was used to induce lipotoxicity damage. Cell condition defined as Vehicle + BSA was established as the control group of the study.

**Metabolic cell viability**—The resazurin reduction assay was used to measure metabolic cell viability. Cells were seeded in 96-well plates and then subjected to the different treatments. After the incubation time, the metabolic cell viability was assessed through the resazurin reduction assay [37]. Briefly, the culture medium was removed, and cells were incubated for 1 h with 80 μL of culture medium supplemented with 10 μg/mL resazurin. The amount of resazurin reduced to resorufin, indicative of metabolic activity, was measured fluorimetrically with 570 nm excitation and 600 nm emission wavelengths in a Biotek Cytation 3 reader (Biotek Instruments, Winooski, VT, USA).

Cell mass measurements—The HepG2 cell line was used. Cells were seeded in 96-well plates and treated with different conditions. Cell mass was determined by measuring cellular protein content [38] using the sulforhodamine B (SRB) assay. For this purpose, cells were washed with 1 x PBS and fixed overnight at 4 °C with 60% TCA. Cells were then stained at 37 ºC for 1 h and washed with 1% acetic acid in water. Finally, 100 μL Tris with a pH of 10 was added, the plates were mixed for 15 min, and the optical density was measured at 540 nm using a Cytation 3 multimode microplate reader (BioTek Instruments, Inc.).

**Intracellular ATP levels**—Cells were seeded in 100 µL of culture medium in white opaque-bottom 96-well plates. After the incubation time, intracellular ATP levels were measured using CellTiter-Glo^®^ Luminescent Cell Viability Assay (Promega Corporation, Madison, WI, USA) following the manufacturer’s instructions [39]. Briefly, 50 µL of culture medium was removed from each well and 50 µL of medium containing CellTiter-Glo^®^ Reagent (CellTiter-Glo^®^ Buffer + CellTiter-Glo^®^ Substrate) was added back to the cells. The contents were mixed for 2 min using an orbital shaker for maximal cell lysis. After 10 min of incubation at 22 °C, the luminescence signal was monitored in a Cytation 3 reader (BioTek Instruments Inc., USA). An ATP calibration curve was produced following the manufacturer’s instructions. The luminescence signal is proportional to the amount of ATP present in the solution.

**Cellular oxygen consumption measurements**—A total of 10,000 cells/100µL/well were seeded in 96-well plates. Oxygen levels at 37 °C were evaluated using a Seahorse XFe96 Extracellular Flux Analyzer (Agilent Scientific Instruments, California, USA). Briefly, one XFe96 sensor per cell plate was incubated overnight at 37 °C with 200 µL/well calibration buffer. Cells were then incubated for 1 h at 37 °C with 175 µL/well of pre-warmed, serum-free, minimal DMEM medium (D5030; Sigma-Aldrich, St. Louis, MO, USA) supplemented with 5 mM glucose, L-glutamine (0.876 g/L; 6 mM), and sodium pyruvate (0.11 g/L; 1 mM). An amount of 25 µL of oligomycin, FCCP, rotenone, and antimycin A solutions were prepared in DMSO, diluted in low-buffered serum-free DMEM medium, and pre-loaded into the ports of each well of the XFe96 sensor. The sensor cartridge and calibration plate were then loaded into the XFe96 Extracellular Flux Analyzer for calibration. An amount of 2 µM oligomycin was injected into reagent delivery port A, 0.5 µM FCPP was injected into port B, and 1 µM rotenone and 1 µM antimycin A were pneumatically injected into reagent delivery port C. Three baseline measurements of oxygen consumption rate (OCR) and extracellular acidification rate (ECAR) of HepG2 for 3 min mix and 5 min measurement cycle were used to measure OCR. The results were normalized to cell mass content at the end of the assay, using the SRB method [38], and analyzed using Wave Desktop 2.6 software.

**Evaluation of neutral lipid content.** After the treatment of the cells under the conditions described above, the accumulation of neutral lipids was examined using the Nile red assay [40]. A 1:200 Nile red solution (0.5 mg/mL stock diluted in acetone) was prepared in a medium without FBS. Cells were washed and incubated with 100 µL of Nile red solution for 1 h to 1.5 h at 37 ºC in the dark. After this time, cells were washed twice with 1 x PBS, and fat content per well was measured fluorometrically at 520 nm excitation wavelength and 620 nm emission wavelength in a Cytation 3 multimode microplate reader (BioTek Instruments, Inc.). The results were normalized to cell mass content at the end of the assay using the SRB method [38].

**Measurement of Catalase activity**—The rate of the decomposition of H_2_O_2_ by catalase was calculated following the method proposed by Grilo et al. [41]. Briefly, 10 mg of frozen liver tissue was homogenized in 50 mM phosphate buffer, pH 7.8. The method was based on the reaction of 10 µg of mitochondria with 50 mM phosphate buffer (pH 7.8) and 10 mM H_2_O_2_ solution in a 96-well plate, being the absorbance measured at 240 nm for 2 min and 30 s in a Cytation 3 multi-mode microplate reader (BioTek Instruments, Inc.). For the positive control, a freshly made solution of catalase (Lot# SLBZ7596, Cat# C1345-1G, Sigma) was used. For the negative controls with catalase inhibitor, a solution of azide (Lot# 058K0146, Cat# S2002-100G, Sigma-Aldrich) was used. Catalase and BSA (Lot# SLCH3826, Cat# A7906-50G, Sigma-Aldrich) were also used in the negative controls. Catalase activity was then calculated and expressed as U/µg of protein.

**Statistics**—Statistical analysis was performed using GraphPad Prism version 8.0.2 (GraphPad Software Inc., San Diego, CA, USA). Data were expressed as box plots, bordered at the 25th and 75th percentiles of the y variable, with a median line at the 50th percentile, showing all independent experiments and presenting all biological replicates. The minimum number of independent experiments performed to assume statistical significance was three. Direct comparisons between two independent groups were made using a Student’s t-test for data that followed a normal distribution and a Mann–Whitney test for non-normal distribution. Multiple comparisons between groups with one independent variable following a normal distribution were made using a one-way ANOVA followed by Tukey’s multiple comparison test, while two-way ANOVA was used for multiple comparisons between groups with more than one independent variable followed by a Dunnett’s multiple comparisons test. Multiple comparisons for non-normally distributed data were performed by using a Kruskal–Wallis test. Significance was accepted with * *p* < 0.05, ** *p* < 0.01, *** *p* < 0.0005, **** *p* < 0.0001.

## 3. Results

**Figure 1:**
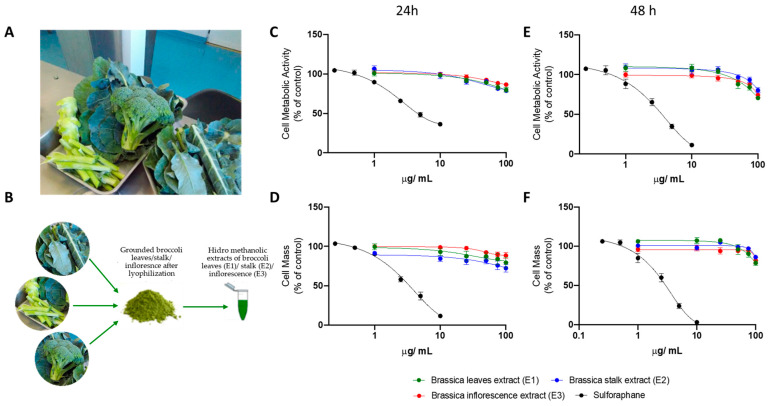
**Cytotoxicity of *Brassica oleracea* var *italica* extracts in HepG2 cells, using different concentrations and for 24 or 48 h.** (**A**) Photo of the plant broken down into leaves, stalks and inflorescences.(**B**) Preparation of broccoli leaf, stalk, and inflorescence extracts. HepG2 cells were seeded and then treated with increasing concentrations of leaf (E1—green), stalk (E2—blue), or inflorescence (E3—red) *Brassica* extracts and sulforaphane (black) for 24 h or 48 and their cytotoxic effects were evaluated through variations in cell metabolic activity (**C**,**E**) and cell mass (**D**,**F**). Data are the mean ± SEM of the three different assays performed, and the results are normalized on the control condition (CTL = 100%). The data obtained with BBP extracts were compared to CTL using a one-way ANOVA with Dunnett’s multiple comparison post-test.

In this study, we evaluated, *in vitro*, the biological potential of broccoli (Figure 1A) BBPs using the human HepG2 cell line subjected to supraphysiological FFAs, which mimics the hepatic pathophysiology observed in NAFLD. Conversely, broccoli leaf, stalk, and inflorescence extracts were prepared (Figure 1B), since they are generally not used for human consumption.

### 3.1. Broccoli Leaf Extracts Possess the Highest Contents of Phenolic Compounds and Antioxidant Capacity

In our broccoli extracts, the leaves presented the highest (*p* < 0.001) content of total phenols, *ortho*-diphenols, and flavonoids and showed higher (*p* < 0.001) antioxidant capacity, for both ABTS and DPPH methods, compared to the stalks and inflorescences (Table 1). In turn, the inflorescences presented a higher (*p* < 0.001) total phenols content and antioxidant activity compared to the stalks (Table 1).

Data are presented as mean (*n* = 3) ± SD. Values for the same parameter evaluated (within the same column) followed by different superscript lowercase letters are significantly different at *p* < 0.001, according to Tukey’s test. DW, dry weight; GA, gallic acid; CAT, catechin; ABTS, 2,2-azinobis (3-ethylbenzothiazoline-6-sulfonic acid) diammonium salt; DPPH, 2,2-diphenyl-1-picrylhydrazyl.

### 3.2. Broccoli Extracts Reduce the Viability of Human Hepatocyte (HepG2) Cells in a Dose-Dependent Manner

HepG2 cells were seeded at a density of 6.0 x 10^4^ cells/cm^2^ and cultured for 24 h. Next, cells were incubated with increasing concentrations of *broccoli* extracts—leaf (E1), stalk (E2), and inflorescence (E3)—for 24 (Figure 1C,D) and 48 h (Figure 1E,F). Sulforaphane was also used as a control. Cell viability, measured through alterations in cell metabolic activity (Figure 1C,E) and cell mass (Figure 1D,F) decreased in the presence of broccoli extracts in a dose-dependent manner. Although no differences were observed between the different *broccoli* extracts, sulforaphane induced HepG2 cell death with higher potency.

### 3.3. A Non-Toxic Concentration of Brassica Oleracea Extracts Had No Effect on Mitochondrial Oxygen Consumption Rates in HepG2 Cells

**Figure 2:**
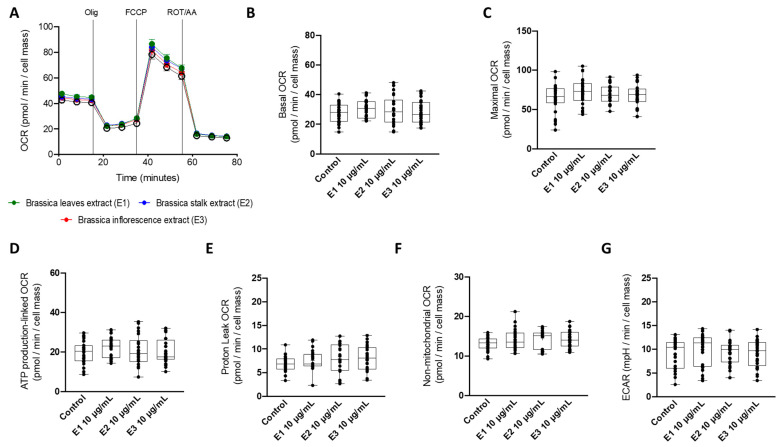
**Effect of broccoli extracts on mitochondrial function of HepG2 cells.** (**A**) Representative image of oxygen consumption rate (OCR) measurement in HepG2 cells treated with vehicle (CTL) or leaf (E1—green), stalk (E2—blue), or inflorescence (E3—red) *Brassica* extracts for 48 h. Several OCR parameters were evaluated: (**B**) cellular basal OCR; (**C**) maximal OCR; (**D**) ATP-production-linked OCR; (**E**) proton-leak-linked OCR; (**F**) non-mitochondrial OCR; and (**G**) basal extracellular acidification (ECAR). Data are the mean ± SEM of 3 independent experiments, and the results are expressed as pmol O_2_/min/cell mass for OCR and mpH/min/cell mass for ECAR. The data obtained with BBP extracts were compared to CTL using a one-way ANOVA with Dunnett’s multiple comparison post-test.

The early identification of new molecules, extracts, or drug-induced mitochondrial function perturbance is of significant importance to avoid attrition in later stages of drug development. As measuring mitochondrial oxygen consumption is one of the most informative ways of assessing mitochondrial dysfunction, we next evaluated the effects of *broccoli* extracts (10 μg/mL; 24 h) on HepG2 mitochondrial oxygen consumption rates (OCR) (Figure 2A). Neither *broccoli* leaf (E1), stalk (E2), nor inflorescence (E3) extracts induced changes in mitochondrial OCR, as demonstrated by a non-significant alteration of basal (Figure 2B), maximal (Figure 2C), and ATP production-linked (Figure 2D), proton leak-related (Figure 2E), and non-mitochondrial (Figure 2F) OCR. Similarly, basal extracellular acidification rate (ECAR) was also not affected (Figure 2G).

### 3.4. A Non-Toxic Concentration of Broccoli Extracts Was Ineffective to Prevent Tert-Butyl Hydroperoxide (t-BHP)-Induced Cell Damage

*Brassica oleracea* by-products are well-known to have compounds presenting antioxidant effects, such as phenols, glucosinolates, carotenoids, and ascorbic acid [27]. Consequently, we evaluated the antioxidant effect of broccoli extracts on t-BHP-induced oxidative damage. First, HepG2 cells were incubated with increasing concentrations of t-BHP for 3 h and cell viability was measured through variations in cell metabolic activity and mass (Figure S1A). t-BHP dose-dependently decreased the cell viability of HepG2 cells and 100 µM was chosen for further testing the antioxidant effects of broccoli extracts. Next, the antioxidant effects of broccoli extracts were tested using two different strategies: (A) HepG2 cells were incubated simultaneously with the extracts and t-BHP for 3 h (co-incubation), and (B) HepG2 cells were incubated with the extracts for 24 h before treatment with t-BHP for 3 h (pre-incubation). In the co-incubation strategy (Figure S1B–D), HepG2 cell viability was reduced by about 50% in cells subjected to t-BHP stimulus (100 μM, 3h). Neither broccoli leaf (E1) (Figure S1B), stalk (E2), (Figure S1C) nor inflorescence (E3) (Figure S1D) extracts were able to prevent t-BHP-induced oxidative stress damage. Similarly, in the pre-incubation strategy (Figure S1E–G), HepG2 cell viability was reduced by about 30% in cells subjected to t-BHP stimulus (100 μM, 3 h), but none of the broccoli leaf (E1) (Figure S1E), stalk (E2) (Figure S1F), or inflorescence (E3) (Figure S1G) extracts were capable to prevent t-BHP-induced oxidative stress damage.

### 3.5. Broccoli Extracts Decreased the Accumulation of Lipid Droplets under Supra-Physiological Concentrations of FA in Human HepG2 Cells

**Figure 3:**
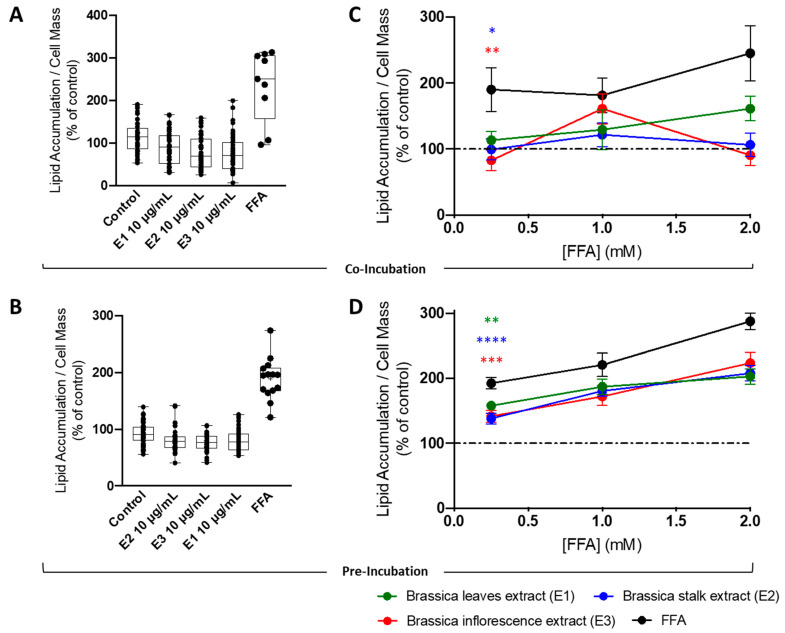
**Effect of broccoli extracts on the accumulation of lipid droplets under supra-physiological concentrations of fatty acids (FA) in human HepG2 cells**. Lipid accumulation in HepG2 cells treated with *Brassica* extracts (leaves—E1, stalks—E2, and inflorescences—E3) for (**A**) 24 h or (**B**) 48 h. Free fatty acids (250 uM; 24 h or 48 h) were used as a positive control. The effect of increasing concentrations of free fatty acids on lipid accumulation in the absence/presence of BBP extracts (E1, E2, and E3). BBP extracts were (**C**) co-incubated or (**D**) pre-incubated with increasing concentrations of FFAs and neutral lipid accumulation was measured through variations in Nile red fluorescence. Data are the mean ± SEM of four different assays performed, and the results were normalized on the control condition (CTL = 100%). The data obtained with BBP extracts were compared to CTL using a one-way ANOVA with Dunnett’s multiple comparison post-test. Significant differences between the indicated conditions are marked by * (*p <* 0.05), ** (*p <* 0.01), *** (*p* < 0.0005), **** (*p* < 0.0001).

The biological potential of *Brassica oleracea* by-products (BBPs) against metabolic disruption in obesity is very well documented [22]. Consequently, we evaluated the effect of broccoli extracts on fatty-acid-induced lipotoxicity. First, HepG2 cells were incubated with broccoli leaf (E1), stalk (E2), or inflorescence (E3) extracts (10 μg/mL) using co-incubation (Figure 3A) and pre-incubation (Figure 3B) strategies, and the cytotoxic effects of each extract were evaluated through changes in intracellular lipid content using Nile red staining. A supra-physiological concentration of FA (250 μM, 24 h) was used as a control. As expected, FFA treatment significantly increased intracellular neutral lipid content in human HepG2 cells (Figure 3A,B). On the other hand, broccoli extracts (10 μg/mL) did not induce a lipid accumulation in HepG2 cells either using a co-incubation (Figure 3A) or pre-incubation (Figure 3B) strategy. Next, the effect of the extracts on fatty-acid-induced lipotoxicity was tested using co-incubation and pre-incubation strategies. In both co-incubation (Figure 2C) and pre-incubation (Figure 3D) strategies, the FFA treatments significantly increased intracellular neutral lipid content in a dose-dependent manner. Interestingly, broccoli stalk (E2) and inflorescence (E3) extracts were able to significantly prevent lipid-overload-induced cell damage both in co-incubation (Figure 3C) and pre-incubation (Figure 3D) strategies. Leaf extract (E1) was only capable of such prevention in the pre-incubation strategy (Figure 3D).

### 3.6. Supra-Physiological Concentrations of Fatty Acids (FA) Had No Impact on Intracellular ATP Levels

**Figure 4:**
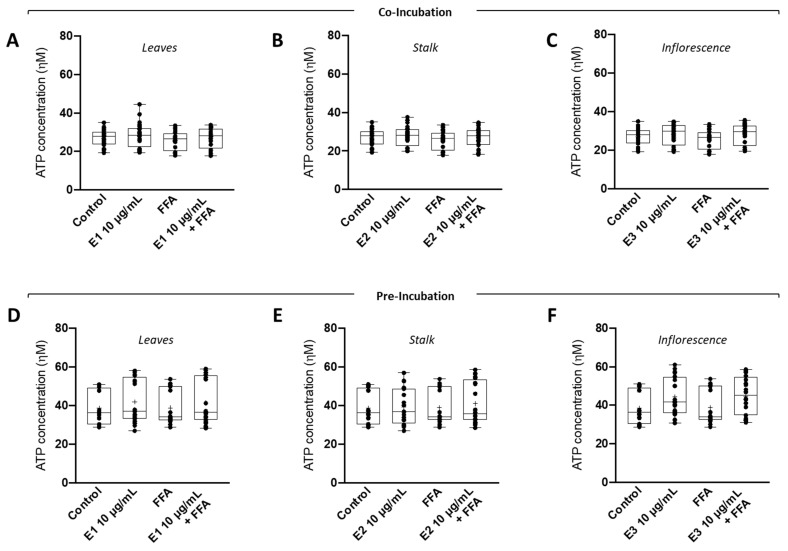
**Effect of broccoli extracts on intracellular ATP levels under supra-physiological concentrations of fatty acids (FA) in human HepG2 cells**. HepG2 cells were (**A**–**C**) co-incubated or (**D**–**F**) pre-incubated with BBP extracts ((**A**,**D**) leaves—E1, (**B**,**E**) stalks—E2, or (**C**,**F**) inflorescences—E3) and then subjected to supraphysiological concentrations of free fatty acids. Intracellular ATP levels were measured using the CellTiter-Glo^®^ Luminescent Cell Viability Assay. Data are the mean ± SEM of four different assays performed, and the results were normalized on the control condition (CTL = 100%). The data obtained with BBP extracts were compared to CTL using a one-way ANOVA with Dunnett’s multiple comparison post-test.

In most mammalian cells, cellular energy in the form of ATP is generated by the integrated action of the glycolysis pathway in the cytosol and the tricarboxylic acid (TCA) cycle and oxidative phosphorylation (OXPHOS) system in the mitochondrion. Next, we evaluated the effect of broccoli extracts on fatty-acid-induced lipotoxicity by measuring intracellular ATP levels. In both co-incubation (Figure 4A–C) and pre-incubation (Figure 4D–F) strategies, no changes were observed in the intracellular ATP levels of FFA-treated HepG2 cells. Similarly, no changes were observed in the intracellular ATP levels of HepG2 cells treated with FFAs in the presence of broccoli leaf (E1), stalk (E2), or inflorescence (E3) extracts. This suggests that the increase in intracellular lipid droplets did not significantly alter intracellular ATP levels, probably because, in this treatment, cells can still supply ATP demands through glycolysis.

### 3.7. Broccoli Extracts Prevented the FFA-Induced Decrease in Catalase Activity in HepG2 Cells

**Figure 5:**
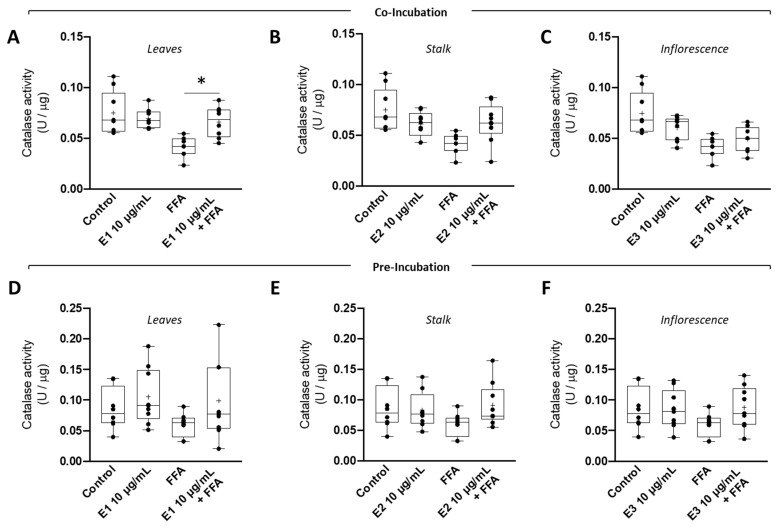
**Effect of broccoli extracts on the activity of antioxidant enzyme catalase under supra-physiological concentrations of fatty acids (FA) in human HepG2 cells**. HepG2 cells were (**A**–**C**) co-incubated or (**D**–**F**) pre-incubated with BBP extracts ((**A**,**D**) leaves—E1, (**B**) and (**E**) stalks—E2, or (**C**,**F**) inflorescences—E3) and then subjected to supraphysiological concentrations of free fatty acids. Catalase activity was assessed in HepG2 cells through the capacity to decompose H_2_O_2_. Data are the mean ± SEM of four different assays performed, and the results were normalized on the control condition (CTL = 100%). The data obtained with BBP extracts were compared to CTL using a one-way ANOVA with Dunnett’s multiple comparison post-test. Significant differences between the indicated conditions are marked by * (*p* < 0.05).

Supra-physiological levels of fatty acids are often correlated with increased ROS levels and inversely correlated with the antioxidant defense system. Consequently, the effect of *Brassica oleracea* extracts on the antioxidant defense system, namely catalase activity, of human hepatoma cells (HepG2) incubated with supraphysiological concentrations of FFAs was also evaluated. In both co-incubation (Figure 5A–C) and pre-incubation (Figure 5D–F) strategies, FFA treatment significantly decreased the catalase activity of HepG2 cells. Interestingly, the co-incubation of broccoli leaf (E1) (Figure 5A) extract was able to significantly prevent lipid-overload-induced decreases in catalase activity. On the other hand, stalk (E2) (Figure 5B,E) and inflorescence (E3) (Figure 5C,F) extracts were not able to prevent the decrease in catalase activity induced by FFA treatment in HepG2 cells. Pre-incubation with E1 also failed to prevent the decrease in catalase activity.

## 4. Discussion

Non-alcoholic fatty liver disease (NAFLD) involves an excessive accumulation of fatty acids in the liver without significant alcohol consumption [8,9]. In NAFLD pathophysiology, hepatocyte mitochondria will up-regulate the β-oxidation pathway to counteract the excessive fatty acid influx, generating reactive oxygen species (ROS) as a by-product [8,9] and ultimately triggering oxidative stress in later stages of the disease [15]. Mitochondria can also become impaired due to excessive triglyceride accumulation in the cytoplasm of hepatocytes in the form of fat droplets [17]. The accumulation may cause an increase in fat oxidation through the peroxisomal pathway, generating further excess ROS and disturbing peroxisomal redox homeostasis [17]. Thus, counteracting oxidative stress and mitochondrial and peroxisomal dysfunction is an appealing approach in the NAFLD context. Although several natural products are also being tested to prevent and/or delay NAFLD progression [42], and despite the number of ongoing clinical trials, there is no approved therapy for NAFLD. In the field of the agricultural industry, a lot of organic waste is generated, as only a small part of the harvested plant is deemed edible for humans and goes on to be sold. The remaining parts of the plant are disposed of using, in many cases, not environmentally friendly methods. *Brassica oleracea* var *italica* is the perfect example. To prevent these negative side effects of broccoli harvesting, the discovery of biomedical value for future applications was proposed. Here, we investigated the medical potential of BBP extracts against steatosis and oxidative damage in human HepG2 cells, chosen as a biological model for hepatocytes, mimicking the hepatic pathophysiology observed in NAFLD.

Broccoli has the highest content of total phenols and flavonoids among *Brassica oleracea* species [20,21], which is linked to the high antioxidant potential of this crop. In our study, among the three extracts, the leaves were the part with the highest phenolic content and antioxidant capacity, which agrees with a previous study from Hwang and collaborators, showing that leaves from different cultivars of broccoli had the highest total phenolic contents and antioxidant activities, followed by inflorescences and then stalks [43]. The data of the present study show that although broccoli extracts displayed dose-dependent toxicity in HepG2 cells, the maximal non-toxic concentration (10 µg/mL) revealed no impact on mitochondrial oxygen consumption rates. Moreover, despite the different broccoli extracts being ineffective in counteracting t-BHP-induced oxidative cell damage, they significantly decreased the accumulation of lipid droplets under supra-physiological concentrations of fatty acids in human HepG2 cells. Although the intracellular levels were not affected in fatty-acid-treated cells, catalase activity was significantly diminished. Interestingly, broccoli extracts, in particular E1 (leaves), significantly prevented the FFA-induced reduction in catalase activity. This potential antioxidant effect was not observed in the experiments with t-BHP, probably due to its more aggressive and extensive mechanisms of induction of ROS [44,45]. The absence of alterations in intracellular ATP levels suggests that the increase in intracellular lipid droplets did not significantly alter intracellular ATP levels, probably because in this treatment, cells can still supply ATP demands through glycolysis. These data agree with our previous observations on the effects of supra-physiological concentrations of FA on HepG2 metabolism [46]. Stalks and leaves are the most studied and characterized of the three extracts since they are the ones with less commercial value and therefore the ones that need value. Under fall season conditions, when the plants that originated the extracts were harvested, *Brassica oleracea* L. has been shown to possess a greater quantity of glucosinolates and myrosinase activity in the stalk than in the leaves [47]. During the fall and winter, temperatures and light exposure periods are lower and there is easier access to water. These two factors, however, are independent of each other, as no correlation between the two has been found [48], meaning that a higher concentration of glucosinolates does not necessarily cause a higher activity of myrosinase, for example. Following the hypothesis that sulforaphane or other isothiocyanate is responsible for the effects observed, increased glucosinolate content and higher myrosinase activity may explain the better results obtained with stalk extract (E2) than with leaf extract (E1). In the available literature, inflorescence extract (E3) has been shown to have superior glucosinolate content than E1 but lower than E2 [49]. However, E3 performed similarly to E2. This means that although it could be argued that glucosinolate content is a factor in the tested properties of the BBPs (the extract with the lowest concentration performed the poorest), it may not be the only one. Myrosinase has been shown to be less active in *Brassica* plants as the plant matures [50]. Since the inflorescence is in the less mature stage of all three extracts, myrosinase activity might make up for the low glucosinolate content. However, both glucosinolate content and myrosinase activity are highly dependent on culture conditions (light, type of agriculture, available water, temperature), so all these explanations are merely hypotheses to explain the obtained results.

The detrimental effect of the excess accumulation of lipids and their derivatives is related to NAFLD. The Nrf2-related antioxidant system has been verified to play key roles in promoting oxidative stress in NAFLD. Nrf2 activation could effectively ameliorate NAFLD-induced inflammation, serum and intrahepatic lipid levels, and pathological changes in the liver *in vitro* and *in vivo*, which indicates that Nrf2 is a promising target for NAFLD therapy. Bioactive antioxidants in various fruits, vegetables, and plants have been shown to protect against NAFLD. In fact, broccoli floret supplementation improves insulin sensitivity and alters the gut microbiome population in a steatotic mice model induced by a high-fat diet [51]. Moreover, dietary broccoli protects against fatty liver development but not against the progression of liver cancer in mice pretreated with diethylnitrosamine [52]. More, broccoli ameliorates NAFLD by increasing lipolysis and promoting liver macrophage polarization toward M2-type. Sulforaphane, a phytochemical isolated from broccoli extracts, induces the classical Nrf2 target enzyme NQO1 [25]. Sulforaphane is a hydrolytic product of the S-β-thioglucoside Nhydroxysulfate (glucosinolate) glucoraphanin. Sulforaphane is produced upon plant tissue injury when a β-thioglucosidase enzyme known as myrosinase comes into contact with its substrate, glucoraphanin.

## 5. Conclusions

Our study shows, *in vitro*, the potential of BBP extracts in reducing lipid accumulation in hepatic cells subjected to supraphysiological concentrations of fatty acids. The effects identified highlight the therapeutic potential of BBPs for the prevention/treatment of NAFLD. In future works, aimed at improving upon and further validating the results here obtained, glucosinolate contents and myrosinase activity will be determined in the extracts used in the experiment. Plant extracts from the same species but derived from plants from different countries and collected during different seasons should also be tested to obtain further information on inter-regional and temporal differences. Furthermore, one *in vivo* proof-of-concept with an NAFLD animal model should be used to test the effects of BBPs and demonstrate their hepatoprotective activity.

## Figures and Tables

**Table 1:** Total phenols (mg GA/g DW), ortho-diphenols (mg GA/g DW), and flavonoids (mg CAT/g DW) content and antioxidant activity (mmol Trolox/g DW) of broccoli leaves, stalks, and inflorescences.

	Phenolic Content			Antioxidant Activity	
Broccoli Part	Total Phenols	*Ortho*-Diphenols	Flavonoids	ABTS	DPPH
**Leaf**	12.35 ± 1.27 ^c^	54.54 ± 6.51 ^b^	8.32 ± 1.25 ^b^	0.0192 ± 0.0089 ^c^	0.0269 ± 0.0067 ^c^
**Stalk**	3.24 ± 0.51 ^a^	4.34 ± 0.81 ^a^	0.84 ± 0.19 ^a^	0.0035 ± 0.0005 ^a^	0.0061 ± 0.0014 ^a^
**Inflorescence**	6.65 ± 1.73 ^b^	7.97 ± 2.17 ^a^	1.30 ± 0.36 ^a^	0.0070 ± 0.0010 ^b^	0.0123 ± 0.0025 ^b^

a, b, c Means with different letters on the same column are significantly different (one-way ANOVA, *p* < 0.05).

## Data Availability

Datasets will be provided upon reasonable request.

## Supplementary Materials

The following supporting information can be downloaded at: https://www.mdpi.com/article/10.3390/nu15040924/s1, Figure S1: Antioxidant effect of *Brassica* oleracea extracts on tert-butyl hydroperoxide (t-BHP)-induced cell damage; Table S1: Pre- and co-incubation treatment periods of broccoli extract for the assessment of antioxidant and lipoprotective effects.
