# Supplementary material for: Brassica oleracea Var italica by-Products Prevent Lipid Accumulation and Cell Death in a Liver Cell Model of Lipid Toxicity
Source: Nutrients. 2023 Feb 12;15(4):924. doi: 10.3390/nu15040924 (PMC9965884; doi:10.3390/nu15040924)
Supplement: Supplementary file 1 [file nutrients-15-00924-s001.zip › nutrients-2094793-supplementary.pdf]

nutrients

MDPI

Article

Brassica oleracea var *italica* by-products prevent lipid accumulation and cell death in a liver cell model of lipid toxicity

José P. Castelão-Baptista [1], Sara Valente [1], Sara Canário [1], David Oppolzer [2,3], Ana Barros [2,3], Carlos Venâncio [2,3], Tânia Martins [2,3], Luís Antunes [2,3], Vilma A. Sardão [1], Eduardo Rosa [2,3], Paulo J. Oliveira [1,*]

1 CNC – Center for Neuroscience and Cell Biology, UC-Biotech, University of Coimbra, Cantanhede, Portugal
2 Centre for the Research and Technology of Agro-Environmental and Biological Sciences (CITAB), University of Trás-os-Montes and Alto Douro (UTAD), Vila Real, Portugal
3 Inov4Agro - Institute for Innovation, Capacity building and Sustainability of Agri-Food production, UTAD, Vila Real, Portugal
* Correspondence: Paulo J. Oliveira, CNC-Center for Neuroscience and Cell Biology, CIBB - Centre for Innovative Biomedicine and Biotechnology, University of Coimbra, 3004-504 Coimbra, Portugal 3060-197, Portugal. E-mail: pauloliv@cnc.uc.pt

Supplementary Tables

Supplementary Table 1. Pre- and co-incubation treatment periods of broccoli extract for assessment of antioxidant and lipoprotective effects.

	Pre-incubation (hours)	Co-Incubation (hours)
tBHP	0	3
	24	3
	28	3
FFA	0	24
	24	24

Supplementary Figures

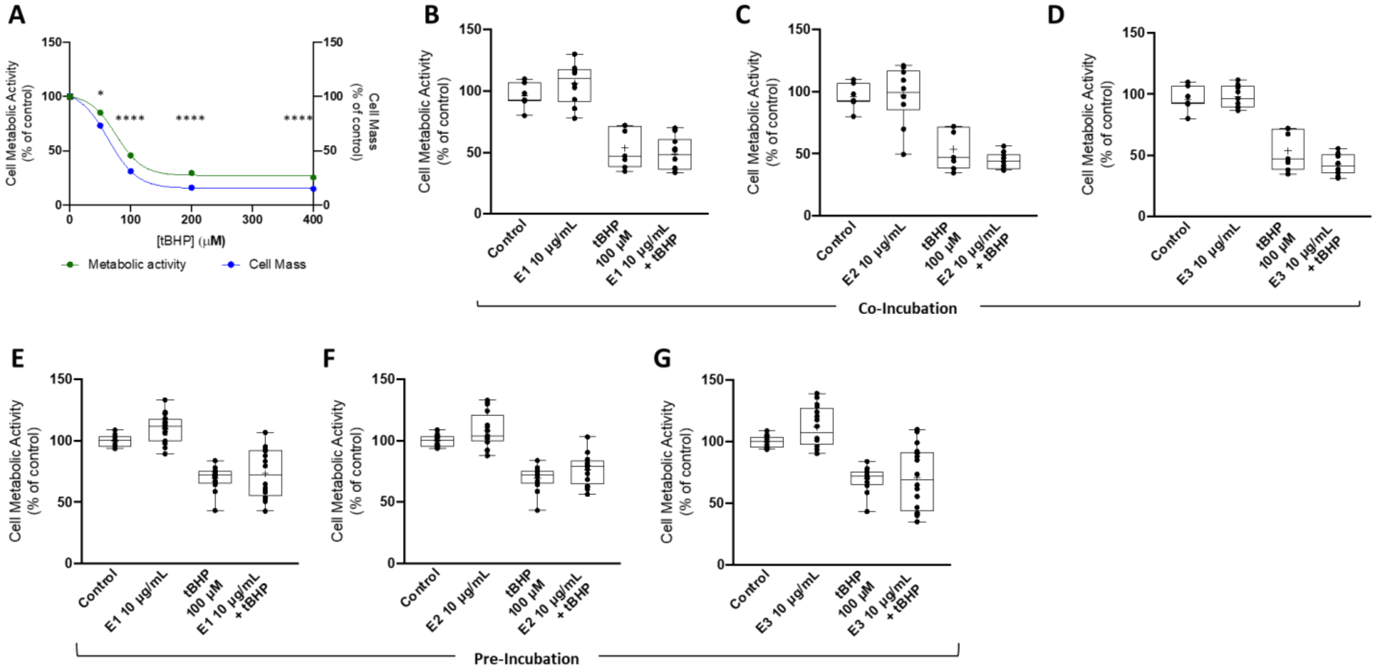

Supplementary Figure 1. Antioxidant effect of Brassica oleracea extracts towards tert-butyl hydroperoxide (tBHP)-induced cell damage. (**A**) HepG2 cells were seeded and then treated with increasing concentrations of t-BHP for 3 h and cytotoxic effects were evaluated through variations in cell metabolic activity and mass. HepG2 cells were also (**B-D**) co-incubated or (**E-G**) pre-incubated with BBP extracts ((**B** and **E**) E1, (**C** and **F**) E2 or (**D** and **G**) E3) and then subjected to t-BHP (100 uM; 3 h) treatment. The antioxidant effects of BBP extracts were evaluated through variations in cell metabolic activity using the resazurin reduction assay. Data are mean ± SEM of four different assays performed, and the results normalized on the control condition (CTL = 100%). The data obtained with BBP extracts was compared to CTL using a one-way ANOVA with Dunnet multiple comparison post-test Significant differences between the indicated conditions are marked by *(p<0.05), ****(p<0.0001).
